# Superficial Siderosis Misdiagnosed As Parkinson’s Disease in a 70-year-old Male Breast Cancer Survivor

**DOI:** 10.7759/cureus.7307

**Published:** 2020-03-18

**Authors:** Stephen J Bordes, Katrina E Bang, R. Shane Tubbs

**Affiliations:** 1 Department of Anatomical Sciences, St. George's University School of Medicine, St. George's, GRD; 2 Department of Anatomical Sciences, St. George's University, St. George's, GRD; 3 Departments of Neurosurgery and Structural & Cellular Biology, Tulane University & Ochsner Clinic Neurosurgery Program, Tulane University School of Medicine, New Orleans, USA

**Keywords:** superficial siderosis, parkinson’s disease, ataxia, bilateral hearing loss

## Abstract

A 70-year-old African American male with a history of hypertension, congestive heart failure, breast cancer status-post six rounds of doxorubicin/cyclophosphamide, and Parkinson’s disease managed with carbidopa/levodopa presented to the emergency department with bilateral hearing loss and ataxia. The patient was admitted and evaluated for possible traumatic, oncological, and pharmacological etiologies. Further investigation revealed hypointensities along the cerebellar folia and basal cisterns on MRI in addition to the two-year history of progressive bilateral hearing loss and gait ataxia. In view of these findings, the patient was diagnosed with superficial siderosis and Parkinson’s medications were discontinued. Superficial siderosis should be considered as a diagnosis in cases of bilateral hearing loss and ataxia in patients with history of anticoagulation and risk factors for prior cerebrovascular accidents or head trauma.

## Introduction

Superficial siderosis is a rare neurological disease associated with chronic subpial deposition of hemosiderin throughout the brain and spinal cord due to recurrent episodes of subarachnoid hemorrhage [1-9]. Disease prevalence within the general population remains unclear, although population-based studies have reported a range of 0.21%-1.43% in patients aged over 55 years, with greater prevalence in those aged over 69 years [2,10]. The disease presents clinically as a triad of bilateral sensorineural hearing loss, ataxia, and myelopathy with pathognomonic findings of hypointensities along the brainstem, cerebellum, and spinal cord identified by multisequence MRI.

In view of the nonspecific sensorineural, neurological, and cerebrovascular findings associated with superficial siderosis combined with its low prevalence within the population, the disease can often be misdiagnosed or missed altogether. Herein, we present a case of superficial siderosis that was misdiagnosed and mistreated as Parkinson’s disease. We suggest that in order to avoid future misdiagnoses, superficial siderosis should be considered as a differential diagnosis for elderly patients, especially those on anticoagulation or with a history of brain trauma or injury, who present with bilateral sensorineural hearing loss and gait ataxia.

## Case presentation

A 70-year-old African American man was admitted to the hospital with bilateral hearing loss and ataxia. The patient was initially brought in by his wife owing to concern for a potential traumatic brain injury, as he had hit his head on a metal gate three days previously while working on his farm. Upon further inquiries concerning history, the patient’s wife stated that his hearing and gait had progressively declined over the previous two years. The patient had first struggled with high pitched sounds, followed by both high- and low-pitched sounds. His wife noticed him sitting closer to his television, struggling to converse in loud settings, and asking others to repeat themselves more frequently. Both the patient and his wife attributed the initial hearing losses to old age.

The patient was diagnosed six years earlier at an outside facility with obstructive sleep apnea, hypertension, benign prostatic hyperplasia, and breast cancer. At that time, he underwent a bilateral mastectomy followed by six months of chemotherapy with doxorubicin and cyclophosphamide. He was subsequently diagnosed with deep vein thrombosis, pulmonary embolism, and congestive heart failure attributed to the chemotherapy. Long-term anticoagulation with warfarin was initiated. The remainder of his medications included lisinopril, metoprolol, amlodipine, tamsulosin, anastrozole, and tamoxifen. The patient followed up consistently with his primary physician and oncologist. At an appointment two years earlier, his gait was noted to be in decline with a tendency to lose balance, and his movements were slowed. He was inappropriately diagnosed with Parkinson’s disease, and carbidopa/levodopa therapy was initiated due to the similarity of physical manifestations between the patient’s presentation and the misdiagnosed movement disorder.

**Figure 1:**
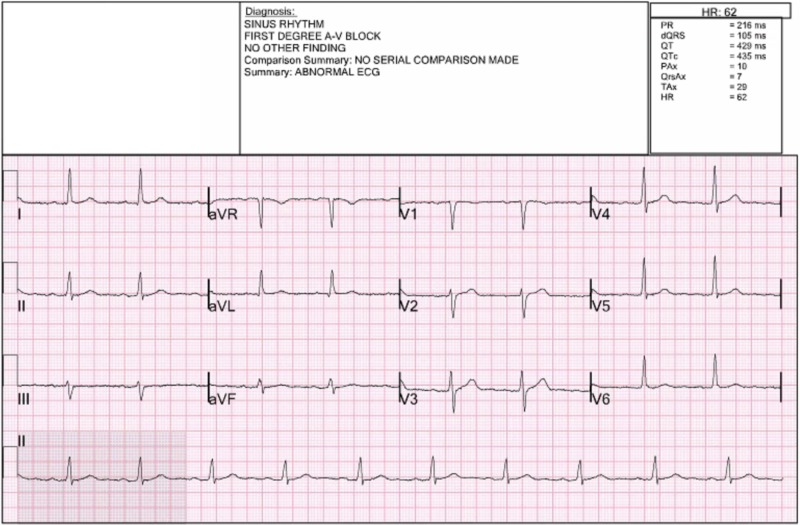
Twelve-lead ECG HR: heart rate; A-V: atrioventricular; ECG: electrocardiogram; Ax; axis Remaining abbreviations cannot be expanded and refer to ECG waves and intervals.

Presently, exam findings on presentation were unremarkable with the exception of bilateral sensorineural hearing loss, ataxia, and 1+ pitting edema to the anterior tibia bilaterally. Weber testing showed no lateralization and Rinne testing revealed air conduction greater than bone conduction. The patient had no signs of trauma or infection to the external ear or tympanum bilaterally. There was no evidence of a bulging membrane. The light reflex was observed. Cranial nerves (CNs) II-XII were otherwise grossly intact. The patient’s gait was markedly ataxic and spastic with a tendency to fall. Romberg testing was negative. He did not exhibit a resting tremor or cogwheeling of the extremities. Head CT without contrast was ordered, which showed no acute intracranial abnormalities; however, old bilateral basal ganglia infarcts were noted. Twelve-lead ECG showed normal sinus rhythm with a first-degree atrioventricular block (Figure 1).

**Figure 2:**
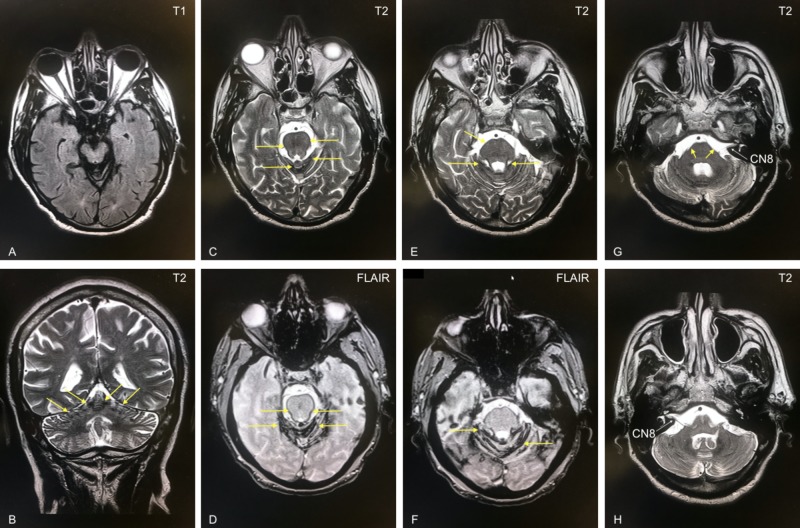
Brain MRI Axial plane multisequence T1, T2, and FLAIR MRI showing the midbrain (A), pons (C-F), and cerebellum (G, H). Coronal plane T2-weighted MRI (B). Hemosiderin hypointensities (black) noted along the cerebellar folia and basal cisterns in both coronal and axial planes (yellow arrows). Cranial nerve VIII (CN8) noted (white arrows). FLAIR, fluid-attenuated inversion recovery

Multisequence brain MRI showed no signs of acute stroke; however, old blood products consistent with the hypointensity of hemosiderin were noted along the cerebellar folia and basal cisterns (Figure 2).

Periventricular small vessel disease and parenchymal atrophy were also noted. Carotid artery and renal ultrasounds showed no evidence of occlusion, obstruction, plaque deposition, or hydronephrosis. Transthoracic echocardiography showed an ejection fraction of 60%, trivial mitral regurgitation, and physiological pulmonary regurgitation (Table 1). There was no sign of ventricular abnormality or dysfunction.

**Table 1:** Transthoracic echocardiography results The findings showed mild mitral annular calcification, trivial mitral regurgitation, normal left ventricle structure and function. TTE: transthoracic echocardiogram; EF: ejection fraction; LV: left ventricle; PW: posterior wall; FS: fractional shortening; EDV: end diastolic volume; ESV: end systolic volume; AO: aorta; LA: left atrium; MV: mitral valve; RAP: right atrial pressure; TR: tricuspid regurgitation; AV: aortic valve; RVSP: right ventricular systolic pressure; PV: pulmonic valve; LVOT: left ventricular outflow tract; VTI: velocity time integral; PASP: pulmonary artery systolic pressure

EF Findings		M-Mode/2D Measurements and Calculations				Doppler Measurements and Calculations			
EF calculated	60.2%	LV diastolic dimension	5.45 cm	LV systolic dimension	4.01 cm	MV peak E-wave	0.51 m/s	AV peak velocity	1.22 m/s
		LV PW diastolic	1.02 cm	LV volume systolic	64.5 mL	MV peak A-wave	0.58 m/s	AV peak gradient	5.95 mmHg
		Septum diastolic	0.87 cm	AO root dimension	3.7 cm	MV E/A ratio	0.89	Estimated RVSP	22 mmHg
		LV volume diastolic	162 mL	LA/aorta	0.86	MV peak gradient	1.06 mmHg	PV peak velocity	1.14 m/s
		LV FS	26.42%	LA dimension	3.2 cm	MV deceleration time	197 ms	PV peak gradient	5.2 mmHg
		LV EDV/LV EDV index	162 mL/69 m^2^			MV E’ velocity	0.06 m/s	LVOT VTI	13.5 cm
		LV ESV/LV ESV index	64.5 mL/27 m^2^			Estimated RAP	10 mmHg	Estimated PASP	21.56 mmHg
						TR velocity	1.70 m/s		
						TR gradient	11.56 mmHg		

Initial laboratory studies can be found in Table 2.

**Table 2:** Laboratory results H: high value (above normal limits); L: low value (below normal limits); WBC: white blood cell count; RBC: red blood cell count; Hgb: hemoglobin; Hct: hematocrit; MCV: mean corpuscular volume; MCH: mean corpuscular hemoglobin; MCHC: mean corpuscular hemoglobin concentration; RDW: red cell distribution width; PT: prothrombin time; INR: international normalized ratio; APTT: activated partial thromboplastin time; CO_2_: carbon dioxide; Est: estimated; HbA_1c_: hemoglobin A_1c_; LDL: low-density lipoprotein; HDL: high-density lipoprotein; THC: tetrahydrocannabinol; Ig: immunoglobulin; Ab: antibody; Ag: antigen; Gen: generation

Hematology		Chemistry		Urine Analysis		Toxicology		Serology		Coagulation	
WBC	5.4	Sodium	143	Collection type	Midstream	Urine opiates	Negative <300 ng/mL	Syphilis IgG/IgM Ab	Nonreactive	PT	14.8 H
RBC	4.35 L	Potassium	4.0	Color	Yellow	Urine barbiturates	Negative <200 ng/mL	HIV 1,2 Ag/Ab, 4thGen	Nonreactive	INR	1.2
Hgb	13.5	Chloride	102	Appearance	Clear	Urine phencyclidine	Negative <25 ng/mL	Lyme Ab	Nonreactive	APTT	28.3
Hct	40.3 L	CO_2_	32	Urine pH	6.5	Urine amphetamines	Negative <1000 ng/mL				
MCV	94	BUN	25 H	Specific gravity	1.013	Urine cocaine	Negative <300 ng/mL				
MCH	31.7	Creatinine	1.8 H	Protein	Negative	Urine THC	Negative <50 ng/mL				
MCHC	33.6	Est GFR	37	Glucose	Negative	Plasma/serum alcohol	<0.010				
RDW	12	Random glucose	115	Occult blood	Negative						
Platelet count	211	HbA_1C_	5.5	Nitrite	Negative						
Immature granulocyte, %	0	Calcium	9.4	Bilirubin	Negative						
Neutrophil, %	54	Phosphorus	4.0	Acetone	Negative						
Lymphocyte, %	35	Magnesium	2.0	Leukocyte esterase	Negative						
Monocyte, %	8 H	Creatinine kinase	226	Osmolality	414						
Eosinophil, %	2	Troponin	<0.30	Random creatinine	130.2						
Basophil, %	1	Triglycerides	147	Random sodium	68						
Nucleated RBC	0	Cholesterol	169	Random potassium	30						
Absolute neutrophils	2.9	LDL	121 H	Random chloride	31						
		HDL	34 L	Random urea nitrogen	665						
		L-Lactate	1.8								

The patient was found to have a kidney injury which was probably chronic and secondary to his hypertension. Elevated prothrombin time was attributable to warfarin; however, the international normalized ratio was subtherapeutic. First-degree atrioventricular block on ECG was attributed to beta-blocker compliance and the pedal edema to amlodipine, a dihydropyridine calcium-channel blocker. Neurology recommended a five-day trial of prednisone in case the symptoms were secondary to an autoimmune or inflammatory etiology; however, the patient exhibited no response and MRI results suggested an alternative diagnosis.

The patient was discharged following a negative stroke and traumatic head injury work-up with an outpatient audiogram appointment. At time of discharge and otolaryngology follow-up visit, the diagnosis was consistent with superficial siderosis. The patient did not meet Parkinson’s criteria, and he was instructed to discontinue carbidopa/levodopa.

## Discussion

Superficial siderosis is a rare and frequently neglected cause of sensorineural hearing loss and progressive ataxia in the elderly [3,4,9]. It develops secondary to slow and repeated intracranial hemorrhages into the subarachnoid space, which result in chronic intra- and extracellular hemosiderin deposition in the subpial layers of the brain, spinal cord, and CNs [3,6,7,9]. Possible causes of these bleeds include intracranial tumors, head trauma, arteriovenous malformations, aneurysms, cervical root avulsion, neurosurgical procedures, brachial plexus injury, amyloid angiopathy, and chronic subdural hematomas [6-8]. Hemosiderin is most commonly found surrounding the brain stem, cerebellum, and basal cisterns as it pools in the posterior fossa, although superficial cortical deposition can be seen as well [1,5,9]. The diagnostic procedure of choice is T2 and susceptibility-weighted (SW) MRI, which visualizes paramagnetic blood products as hypointense [5,9]. T1 and gradient echo MRI are less sensitive in detecting blood products [5]. CT is not ideal for detecting hemosiderin, but these scans can be used to detect hemosiderin as hyperintense or to rule out other substances such as calcium that can appear as hypointense on T2 and SW MRI [9].

Superficial siderosis preferentially disturbs tissues with greater exposure to CSF and longer glial segments. This increases the rate of iron overload and subsequent lipid peroxidation of surrounding structures [3]. The condition has an atypical triad of impairment, which includes bilateral sensorineural hearing loss owing to the nature and long time course of CN VIII injury around the basal cisterns (Figure 2); gait ataxia due to involvement of the cerebellar folia and vermis (Figure 2); and myelopathy due to pyramidal tract involvement [3,8,9]. Other symptoms can include anosmia, which is often overlooked owing to the long CN I glial sheath; dementia due to cortical necrosis; cerebral atrophy; and declining executive function [3,9].

Hearing loss in this age group is often attributed to presbycusis, and further investigation is curtailed [4]. However, the presence of additional symptoms should raise a strong clinical suspicion for an alternative ongoing disease process. Differential diagnoses should include multiple sclerosis, autoimmune disease, neurosyphilis, Lyme disease, ototoxic pharmaceuticals (salicylates, aminoglycosides, platinum-based chemotherapeutic agents, etc.), and superficial siderosis [4,7]. It is less likely for Parkinson’s disease to be confused with siderosis. We recommend increasing suspicion for individuals on anticoagulation and individuals with prior head injuries.

Bleeding sources are identified in less than 50% of cases [4,9]. Management is approached stepwise, with a primary goal of stopping the bleeding, and secondary goals focused on chelation of hemosiderin deposits with lipid-soluble agents such as deferoxamine, deferiprone, and trientine. However, the risks often outweigh the benefits as symptomatic improvement can be negligible [7,8].

## Conclusions

Superficial siderosis should be considered in any case of progressive bilateral sensorineural hearing loss and ataxia with or without additional accompanying symptoms, especially in individuals on long-term anticoagulation or those with prior head injuries. It is commonly misdiagnosed or underdiagnosed owing to its low frequency of occurrence in the general population. Treatment entails surgical correction of the hemorrhage, followed by iron chelation should benefits outweigh risks. We recommend increasing clinical sensitivity in order to decrease time to diagnosis and improve overall patient outcomes.
